# Rethinking Mild Therapeutic Hypothermia After Out-of-Hospital Cardiac Arrest: Dead End or Missed Opportunity? An Expert Viewpoint

**DOI:** 10.3390/jcm15218676

**Published:** 2026-11-06

**Authors:** Julia M. Umińska, Jakub Ratajczak, Piotr Niezgoda, Przemysław Żuratyński, Dawid Mielczarek, Łukasz Pawłowski, Maciej Chudaś, Przemysław Jasiewicz, Łukasz Szarpak, Klaudiusz Nadolny, Jerzy Robert Ładny, Agnieszka Tycińska, Aldona Kubica, Robert Gajda, Miłosz Jaguszewski, Beata Średniawa, Wiktor Kuliczkowski, Maciej Lesiak, Przemysław Trzeciak, Mariusz Gąsior, Tomasz Mroczek, Janina Stępińska, Marko Noč, Zuzana Motovska, Udaya Tantry, Young-Hoon Jeong, Paul A. Gurbel, Diana A. Gorog, Rahima Gabulova, Salvatore Di Somma, Jolita Badariene, Stefan Frantz, Stefan Störk, Eliano P. Navarese, Jacek Kubica

**Affiliations:** 1 Collegium Medicum, Nicolaus Copernicus University, 85-094 Bydgoszcz, Poland; 2 Staszic Specialist Hospital in Piła, 64-920 Piła, Poland; 3 Institute of Medical Science, The John Paul II Catholic University of Lublin, 20-037 Lublin, Poland; 4 Department of Emergency Medical Service, Faculty of Medicine, Silesian Academy in Katowice, 40-752 Katowice, Poland; 5 Department of Emergency Medicine, Medical University of Bialystok, 15-276 Bialystok, Poland; 6 Department of Cardiology, Medical University of Bialystok, 15-276 Bialystok, Poland; 7 Modern Medical Technologies Center, 87-100 Toruń, Poland; 8 Gajda-Med District Hospital, 06-100 Pułtusk, Poland; 9 First Department of Cardiology, Medical University of Gdansk, 80-210 Gdansk, Poland; 10 Department of Cardiology and Electrotherapy, Faculty of Medical Sciences in Zabrze, Medical University of Silesia Katowice, 41-800 Zabrze, Poland; 11 Institute for Heart Diseases, Wroclaw Medical University, 50-556 Wrocław, Poland; 12 Department and Clinic of Cardiology, University of Medical Sciences, 61-848 Poznań, Poland; 13 Third Department of Cardiology, Faculty of Medical Sciences in Zabrze, Silesian Center for Heart Diseases, Medical University of Silesia in Katowice, 41-800 Zabrze, Poland; 14 Department of Pediatric Cardiac Surgery, University Children’s Hospital, Jagiellonian University Medical College, 30-663 Kraków, Poland; 15 National Institute of Cardiology, 04-628 Warszawa, Poland; 16 International Cardiovascular Center MC Medicor, 6310 Izola, Slovenia; 17 Department of Cardiology, Third Faculty of Medicine Charles University, 100 00 Prague, Czech Republic; 18 University Hospital Královské Vinohrady, 100 34 Prague, Czech Republic; 19 Sinai Center for Thrombosis Research and Drug Development, Sinai Hospital of Baltimore and Lifebridge Health System, Baltimore, MD 21215, USA; 20 CAU Thrombosis and Biomarker Center, Chung-Ang University Gwangmyeong Hospital, Seoul 14353, Republic of Korea; 21 Gwangmyeong and Department of Internal Medicine, Chung-Ang University College of Medicine, Seoul 06973, Republic of Korea; 22 National Heart and Lung Institute, Imperial College, London W12 0NN, UK; 23 Centre for Health Services Research, University of Hertfordshire, Hatfield AL10 9AB, UK; 24 Teaching-Therapeutical Hospital, Azerbaijan Medical University, AZ 1022 Baku, Azerbaijan; 25 GREAT Heath Science Network, 00136 Rome, Italy; 26 Clinic of Cardiovascular Diseases, Faculty of Medicine, Institute of Clinical Medicine, Vilnius University, LT-08661 Vilnius, Lithuania; 27 Department of Clinical Research and Epidemiology, Comprehensive Heart Failure Center, University Hospital Würzburg, 97080 Würzburg, Germany; 28 Department of Internal Medicine I, University Hospital Würzburg, 97080 Würzburg, Germany; 29 Department of Life and Health Sciences, Link Campus University, 00165 Rome, Italy; 30 American Heart of Poland, 64-920 Piła, Poland

**Keywords:** out-of-hospital cardiac arrest, mild therapeutic hypothermia, targeted temperature management

## Abstract

Out-of-hospital cardiac arrest remains associated with high mortality and neurological disability. Mild therapeutic hypothermia has been questioned after large trials failed to demonstrate superiority over controlled normothermia with fever prevention. In this expert viewpoint, we argue that these neutral findings should not be interpreted as proof of biological futility in every patient and setting. The clinical effect of hypothermia may depend on patient phenotype, timing of initiation, cooling technique, temperature-control quality, and integration with contemporary post-resuscitation care. This may be particularly important in patients undergoing percutaneous coronary intervention, in whom hypothermia can impair the pharmacokinetics and antiplatelet effects of oral P2Y12 inhibitors. Current evidence does not support routine hypothermia in all comatose survivors. However, uncertainty remains regarding its value in selected patients. Patients with an intermediate degree of ischemic brain injury may represent a plausible target for neuroprotection, but this remains an unproven hypothesis. Future trials should investigate whether injury severity modifies the effects of early, precisely delivered hypothermia, using standardized protocols, active normothermia as the comparator, and prespecified antithrombotic and post-resuscitation strategies.

## 1. Introduction

Out-of-hospital cardiac arrest (OHCA) remains one of the most dramatic emergencies in cardiovascular medicine, burdened by high mortality and a substantial risk of severe neurological injury among survivors [1,2]. Although advances in emergency medical systems, cardiopulmonary resuscitation, early defibrillation, and post-resuscitation intensive care have improved outcomes in selected populations, the prognosis of many patients continues to depend largely on the quality of treatment delivered after return of spontaneous circulation (ROSC). However, the challenge is not limited to restoring circulation, but to reduce the burden of ischemia–reperfusion injury and preserve the possibility of meaningful neurological recovery [3].

For many years, mild therapeutic hypothermia (MTH), usually targeting a core body temperature of 32–34 °C, was considered one of the few interventions capable of attenuating secondary reperfusion brain injury after OHCA [4,5,6].

However, the publication of the TTM trial, followed by the TTM2 trial comparing hypothermia at 33 °C with normothermia and active fever prevention, profoundly changed opinions regarding MTH [7,8]. These studies were rapidly interpreted as showing that hypothermia was no longer beneficial.

This interpretation has also influenced guideline language. Contemporary recommendations have moved away from routine cooling to 33 °C for all comatose post-arrest patients and now emphasize deliberate temperature control, continuous core temperature monitoring, and active fever prevention [9,10]. Importantly, however, this shift should not be misread as proof that lower temperature targets are biologically irrelevant in every patient.

In our view, such a conclusion is premature. It reflects an understandable desire for clarity, but it overlooks the fact that temperature management after cardiac arrest is not a single, uniform intervention. Its clinical impact is likely to depend on multiple interrelated factors, including the biological timing of cooling, the technique used to achieve and maintain target temperature, the precision of thermal control, the characteristics of the treated population, and the quality of associated post-resuscitation care. Neutral results observed with the strategies tested in these trials do not necessarily establish biological futility of all forms of hypothermia. However, they may reflect either the absence of a clinically meaningful treatment effect or, hypothetically, heterogeneity related to patient phenotype, timing, and intervention delivery.

This expert viewpoint revisits the current role of MTH after OHCA and argues that the question is not closed. Instead of asking whether hypothermia works in all patients, contemporary research should ask whether selected forms of hypothermia, applied with adequate timing and technical precision in carefully defined patients, still offer clinically meaningful benefit.

## 2. The Distinction Between TTM and MTH

A central source of confusion in this field is the tendency to use the terms targeted temperature management (TTM) and mild therapeutic hypothermia (MTH) interchangeably. This is conceptually inaccurate and clinically consequential. TTM is a broad therapeutic framework encompassing deliberate temperature control, including hypothermia and normothermia with fever prevention, whereas MTH refers specifically to intentional cooling to approximately 32–34 °C [9,10]. Although the distinction may appear modest, it should not be dismissed as purely semantic.

In recent guideline documents, the broader concept of targeted temperature management (TTM) is increasingly framed as “temperature control” because the therapeutic objective is not necessarily the induction of hypothermia but the deliberate control of body temperature and prevention of fever [9,10]. Within this framework, mild therapeutic hypothermia (MTH), defined here as intentional cooling to approximately 32–34 °C, represents one specific temperature-control strategy rather than a synonym for TTM. In contrast, active normothermia refers to deliberate maintenance of temperature within the normothermic range using active temperature-control measures, while fever prevention specifically denotes interventions aimed at avoiding or treating temperature above a predefined threshold. These intentional strategies should be distinguished from passive cooling, in which body temperature decreases unintentionally because of environmental or care-related factors, and spontaneous hypothermia, in which temperature decline reflects the patient’s physiological condition and may be associated with severe injury, shock, or impaired thermoregulation.

The biological effects of cooling are temperature dependent. A patient maintained at 33 °C is not exposed to the same physiological intervention as a patient managed at 36 °C or with a normothermia target of ≤37.5 °C and protocolized fever prevention. Hypothermia at lower temperatures reduces cerebral metabolic demand, modulates excitotoxicity, influences inflammatory signalling, affects mitochondrial function, and may alter the kinetics of reperfusion injury more substantially than strategies limited to normothermia [11,12,13].

This distinction is especially important when interpreting contemporary trials. Neither the TTM nor the TTM2 trial compared hypothermia with uncontrolled standard care. The TTM trial compared temperature targets of 33 °C and 36 °C, whereas TTM2 compared hypothermia at 33 °C with normothermia and protocolized fever prevention [7,8]. In the TTM2 normothermia group, the temperature target was ≤37.5 °C; device-based cooling was initiated if the temperature reached ≥37.8 °C, with the device set to 37.5 °C [8]. These are clinically important comparisons, but their findings should not be generalized beyond the strategies actually evaluated. They challenge the universal superiority of one broadly applied temperature strategy over another but do not prove that all MTH approaches lack value in all patients.

## 3. The Biological Rationale for Hypothermia

The case for hypothermia after cardiac arrest was based on the biology of global ischemia and reperfusion injury. During cardiac arrest, interruption of cerebral perfusion leads to the depletion of high-energy phosphates, membrane depolarization, intracellular calcium overload, excitotoxic neurotransmitter release, and early cell damage. Following ROSC, reperfusion is essential for survival, but it also initiates a cascade of secondary injury involving oxidative stress, inflammatory activation, mitochondrial dysfunction, endothelial injury, microcirculatory disturbance, and delayed apoptotic signalling [13,14]. These processes evolve over hours rather than seconds, creating a plausible therapeutic window during which neuroprotective interventions might alter outcome.

Mild hypothermia has long been attractive because it targets several of these pathways simultaneously. It decreases cerebral oxygen demand, suppresses excitotoxicity, reduces free radical generation, modulates inflammatory responses, and may attenuate blood–brain barrier dysfunction and cerebral oedema [13,15]. Importantly, these effects are most biologically plausible when cooling is initiated early enough to influence the evolving reperfusion cascade. Therefore, timing is an important procedural factor of the intervention.

For this reason, the widespread conclusion that hypothermia is ineffective requires caution. It is pivotal whether sufficiently early, precise, and biologically meaningful hypothermic exposure was truly delivered. A therapy with a sound mechanistic basis may fail to show benefit if applied too late, too inconsistently, or in patients in whom the predominant injury pathways are no longer modifiable. Neutral trial results, therefore, do not automatically negate the biological rationale. They may instead indicate that the intervention, as delivered, was not optimal for demonstrating its full potential.

## 4. The Early MTH Trials

The modern era of MTH was shaped by two pivotal randomized studies published in 2002, both of which demonstrated improved neurological outcomes in comatose survivors of OHCA treated with induced hypothermia [5,6]. These trials were transformative because they provided the first convincing clinical evidence that temperature manipulation could improve neurologically meaningful recovery after OHCA. Their results aligned with experimental data and created a coherent rationale for incorporating hypothermia into standard post-resuscitation care.

Over time, those early findings were supported by additional clinical experience, including observational analyses and network-based implementation strategies. Particularly noteworthy was the ACS network-based implementation of MTH, which showed that it could be successfully integrated into a structured invasive cardiology pathway and was associated with improved clinical outcomes in comatose OHCA survivors [16]. This experience suggested that when cooling is embedded within a coordinated system of coronary diagnosis, PCI, critical care, and protocolized post-arrest management, its clinical value may be enhanced.

Moreover, subsequent observational evidence suggested that MTH could still retain prognostic relevance in selected patients, particularly in those with OHCA occurring in the early phase of myocardial infarction [17]. Although such data do not carry the evidentiary weight of large, randomized trials, they support the view that hypothermia cannot be dismissed solely on the basis of a single overarching narrative. The totality of the literature is more heterogeneous, and more nuanced, than the simplified conclusion that “hypothermia no longer works”.

## 5. TTM and TTM2 Changed Practice, but Should Not End the Debate

The TTM trial was a major scientific milestone because it challenged the assumption that lower temperature was necessarily superior. By showing no significant difference between active temperature management at 33 °C and 36 °C, it prompted a fundamental re-evaluation of established practice [7]. The TTM2 trial further advanced this reappraisal by demonstrating no advantage of hypothermia at 33 °C over normothermia with protocolized fever prevention (target ≤ 37.5 °C) in the studied OHCA population [8].

Together, these trials demonstrated that routine cooling to 33 °C does not confer an overall clinical benefit compared with active temperature management at 36 °C or active normothermia with fever prevention in broad populations of comatose OHCA survivors [7,8]. Accordingly, contemporary guidelines emphasize deliberate temperature control, continuous core-temperature monitoring, and active fever prevention rather than the routine use of MTH in all patients [12]. However, these findings should not be interpreted as definitive evidence that hypothermia is biologically ineffective in every clinical context. Such a conclusion would extend beyond the populations, interventions, and temperature-control strategies evaluated in these trials.

Importantly, the major clinical studies of hypothermia and temperature management differed substantially in patient selection, timing of cooling initiation, temperature targets and comparators, cooling techniques, and overall intervention delivery. These differences, together with the principal outcomes and methodological limitations of the key studies, are summarized in Table 1.

Renewed interest in MTH must explicitly incorporate treatment-related harm. Cooling to 33 °C may increase bradyarrhythmias, complicate haemodynamic management, intensify shivering control, prolong sedative exposure, and alter electrolyte, glucose, coagulation, and antiplatelet profiles. Therefore, the clinically relevant question is not simply whether hypothermia can still exert neuroprotection, but whether any potential neuroprotective effect outweighs its physiological cost in a given patient [5,7,18,20,29].

A major reason for caution is that the TTM and TTM2 trials were pragmatic multicentre studies. Such designs improve external validity, but they also introduce variability in intervention delivery. In a technically dependent treatment such as MTH, the timing of cooling initiation relative to ROSC, delay to target temperature, cooling method, temperature monitoring, shivering control, rewarming, and co-interventions may all affect the actual physiological exposure achieved. Accordingly, a neutral average result across a large and heterogeneous trial population does not necessarily exclude the possibility of differential treatment effects across specific patient phenotypes or modes of intervention delivery. This possibility, however, remains hypothesis-generating and requires prospective confirmation.

This distinction is especially important in critical care, where treatment effects may depend on both patient selection and the way an intervention is delivered. Whether MTH represents such a context-dependent intervention remains uncertain and should be addressed in appropriately designed prospective trials.

## 6. Methodological Limitations: Cooling Performance Matters

Cooling method is an important consideration when interpreting temperature-management trials. Analyses of the TTM and TTH48 datasets found that intravascular cooling was associated with more precise temperature control than surface cooling [18,20]. A post hoc analysis of TTM2 also reported faster achievement of target temperature, less time outside the desired range, and less post-treatment fever with intravascular devices [29]. These differences may be relevant when evaluating the thermal exposure actually delivered.

Such findings demonstrate that different cooling methods may not provide identical thermal exposure, but they do not establish that greater temperature precision translates into improved clinical outcomes. Comparisons of intravascular and surface cooling are particularly susceptible to selection bias and residual confounding when cooling technique is not randomized. It therefore remains uncertain whether differences in cooling performance modify the clinical effect of MTH. This possibility should be regarded as a hypothesis requiring prospective evaluation rather than an explanation for the neutral results of previous trials.

### 6.1. Timing: An Inseparable Component of the Intervention

The biological rationale for MTH implies that timing is critical. Hypothermia is intended to mitigate secondary reperfusion injury, and such protection is most plausible when cooling is initiated before the injury cascade becomes self-sustaining [10,13,15]. Consequently, delayed cooling may fail even if the final temperature target is achieved.

This issue has long been recognized. Trials of ultra-rapid cooling were developed precisely because many investigators believed that earlier induction might be essential for meaningful neuroprotection [13,21]. Yet in routine practice, and even in large, randomized studies, delays often occur at multiple levels: between collapse and ROSC, between ROSC and hospital arrival, between arrival and the initiation of cooling, and between initiation and target achievement [7,8,24]. Each of these intervals may influence the biological plausibility of the benefit.

An additional methodological challenge is that temperature may decline before hospital admission even in the absence of an intentional cooling intervention. Environmental exposure, removal of clothing, absence of active warming, administration of non-warmed fluids, prolonged on-scene care, ambulance conditions, and transport duration may all contribute to unintended passive cooling. Moreover, spontaneous hypothermia may reflect severe neurological injury, impaired thermoregulation, prolonged resuscitation, shock, or systemic hypoperfusion rather than a therapeutic effect [23]. Consequently, the temperature recorded at hospital or ICU admission may not adequately characterize the preceding thermal exposure. Future studies should distinguish intended therapeutic cooling from unintended passive cooling and spontaneous hypothermia related to injury severity. Serial core temperature measurements should therefore begin as early as feasible and be interpreted together with ambient conditions, active warming measures, temperature of administered fluids, duration of on-scene care and transport, measurement site, and the complete prehospital time–temperature trajectory.

A distinct situation arises during extracorporeal cardiopulmonary resuscitation (eCPR), in which extracorporeal circulation may produce rapid intentional or unintended cooling, potentially even before ROSC. This exposure differs fundamentally from conventional post-ROSC MTH because eCPR patients typically have prolonged low-flow times, refractory cardiac arrest, and greater haemodynamic severity, while the thermal effects of cooling may be difficult to separate from those of extracorporeal perfusion itself. Accordingly, eCPR-associated cooling should be considered a distinct intervention or co-intervention rather than combined uncritically with conventional MTH. Future studies should prespecify eCPR status and document the timing of extracorporeal flow, temperature trajectory, heat-exchanger use, and relevant neurological and device-related safety outcomes.

At the same time, the literature on cooling speed is complicated by an important paradox. Several studies have suggested that patients with worse neurological outcomes may reach target temperature faster [24,31,32]. This apparently counterintuitive observation likely reflects the fact that faster cooling is not always a marker of better treatment delivery but may also indicate more severe neurological injury and impaired thermoregulatory capacity.

This interpretation is supported by analyses of OHCA patients treated with MTH, in which shorter induction times were associated with poor neurological outcome on univariate analysis, whereas older age and lower admission Glasgow Coma Scale remained the principal independent predictors after adjustment [23]. In the same study, longer induction time was associated with lower lactate levels and shorter CPR duration, suggesting that patients with less severe ischemic injury retain greater resistance to cooling [23]. These findings are consistent with the hypothesis that rapid temperature decline may partly reflect more extensive brain damage rather than a more effective therapeutic intervention.

Therefore, when discussing the importance of timing, a distinction must be made between the early initiation of cooling as a therapeutic goal and rapid target achievement as an observational phenomenon. The first may be biologically beneficial. The second may be confounded by underlying injury severity. Failure to distinguish these concepts has contributed to contradictory interpretations of the literature.

### 6.2. The Heterogeneity of OHCA and the Limits of Universal Conclusions

OHCA is not a single disease entity. It comprises diverse aetiologies, initial rhythms, durations of circulatory arrest, haemodynamic trajectories, and degrees of neurological injury. A witnessed ventricular fibrillation arrest caused by acute coronary occlusion with immediate bystander CPR differs substantially from a prolonged non-shockable arrest with delayed resuscitation and severe multiorgan failure. These differences provide a rationale for investigating whether the response to MTH varies across clinical phenotypes.

Observational studies have repeatedly shown that outcome after MTH is strongly influenced by baseline patient characteristics, including age, initial neurological status, cardiogenic shock, rhythm profile, CPR duration, adrenaline dose, and metabolic parameters such as lactate [31,33]. These variables are central determinants of prognosis and likely of treatment responsiveness.

The severity of ischemic brain injury may be particularly relevant to future tailored temperature-management strategies. Conceptually, patients with an intermediate degree of injury may have the greatest potential for neuroprotection. Patients with minimal injury may have little room for additional benefit, whereas those with extensive irreversible injury may be unlikely to benefit and may be more vulnerable to treatment-related adverse effects. Early assessment could incorporate complementary variables, including no-flow and low-flow duration, neurological examination, pH and lactate, electrophysiology, biomarkers, and neuroimaging. However, no validated thresholds currently allow the reliable assignment of individual patients to a specific temperature strategy early after ROSC. Severity-based tailored temperature management should therefore be regarded as a hypothesis requiring prospective validation rather than a basis for current clinical decision-making.

Evidence concerning the initial cardiac rhythm remains inconsistent. Observational analyses have suggested a potential benefit of MTH in selected patients with shockable rhythms [34]. However, a secondary analysis of TTM2 restricted to a HACA-like population with an initial shockable rhythm found that hypothermia at 33 °C did not improve 6-month survival or functional outcome and was associated with more arrhythmias resulting in haemodynamic compromise [25].

The evidence in patients with an initial non-shockable rhythm is similarly complex. In HYPERION, hypothermia at 33 °C was associated with a higher proportion of patients surviving with a favourable neurological outcome at 90 days than targeted normothermia. However, mortality did not differ significantly, and the absolute number of favourable neurological outcomes was small [22]. A subsequent individual-patient-data meta-analysis combining OHCA patients with an initial non-shockable rhythm from HYPERION and TTM2 found no significant reduction in mortality or improvement in functional outcome with hypothermia at 33 °C [28]. These findings do not establish a reproducible benefit in this rhythm-defined population.

Reanalysis of earlier datasets has suggested a potential benefit when MTH is initiated early and continued across the prehospital and in-hospital phases of care [19]. Other exploratory studies have examined hypothermia in patients with cardiogenic shock, including those receiving VA-ECMO [26,35]. These findings remain insufficient to establish efficacy in specific subgroups. They support further prospective investigation with predefined phenotypes and treatment protocols while preserving a clear distinction between biological plausibility, prognostic associations, and demonstrated treatment benefit.

### 6.3. Antiplatelet Therapy: A Clinically Important Interaction with MTH

In many patients, OHCA occurs in the context of acute coronary syndrome, and coronary angiography with PCI may be an important component of post-resuscitation care [9,16]. The urgency of coronary angiography depends on the clinical presentation. Emergency angiography is recommended in patients with persistent ST-segment elevation and should be considered in those with a high suspicion of ongoing coronary occlusion, particularly in the presence of haemodynamic or electrical instability [9,10]. In patients without ST-segment elevation who do not require emergency angiography, the COACT and TOMAHAWK randomized trials did not demonstrate a survival benefit from routine immediate angiography compared with a delayed or selective strategy [36,37]. These findings should not be extrapolated to patients requiring urgent intervention because of ongoing ischemia or haemodynamic or electrical instability. The interaction between MTH and invasive coronary treatment should therefore be considered within the relevant clinical phenotype.

In patients undergoing PCI after OHCA, treatment requires both coronary reperfusion and effective platelet inhibition during the early period after stenting. Any evaluation of MTH in this setting should therefore consider its interaction with antiplatelet therapy [38].

Clinical studies have reported delayed and reduced ticagrelor exposure in patients with acute myocardial infarction undergoing MTH after OHCA [39]. Total exposure during the first 12 h after loading and peak plasma concentration were lower, while the time to peak concentration was prolonged [39]. Pharmacodynamic studies have also documented delayed or insufficient platelet inhibition in this setting [40,41,42]. However, the contribution of hypothermia is difficult to separate from the effects of cardiac arrest, shock, and critical illness.

These effects may be compounded by opioid administration and impaired gastrointestinal absorption. Morphine, myocardial infarction, gastrointestinal hypoperfusion, and critical illness can delay the absorption and onset of action of oral P2Y12 inhibitors [42,43,44,45,46]. Together, these factors may create a transient period of insufficient platelet inhibition when thrombotic risk after PCI is particularly high.

Whether this pharmacological vulnerability materially affects the net clinical benefit of MTH remains uncertain. The hypothesis that coronary thrombotic complications may have attenuated a potential benefit of hypothermia in selected patients is speculative. It requires prospective evaluation with dedicated assessment of stent thrombosis, bleeding, and antiplatelet treatment strategies.

Intravenous P2Y12 inhibition with cangrelor may provide a bridging strategy in comatose OHCA patients undergoing PCI when immediate platelet inhibition is required and the onset of oral treatment is uncertain [47,48,49,50]. This approach should be considered selectively rather than as a universal recommendation. Future MTH protocols in OHCA related to acute coronary syndrome should prespecify antiplatelet and anticoagulation strategies, routes of administration, timing of loading doses, possible use of platelet-function testing, and safety endpoints, including stent thrombosis and major bleeding.

### 6.4. Technical Issues of MTH

The efficacy of MTH depends not only on the chosen target temperature, but also on the fidelity with which the intervention is implemented. This includes the site of temperature measurement, the response to shivering, the consistency of maintenance, and the rate of rewarming. All of these factors influence whether the patient is truly exposed to the intended physiological intervention.

Temperature monitoring deserves particular attention. In comatose post-arrest patients undergoing MTH, oesophageal and urinary bladder measurements may differ in their ability to reflect rapid changes in core temperature, with oesophageal monitoring appearing more responsive during dynamic phases [11]. Such differences may seem technical, but they have practical and scientific consequences. If measurement methods differ substantially across centres or studies, then the same nominal temperature target may correspond to somewhat different physiological realities. For this reason, future studies should report the delivered thermal dose rather than only the assigned temperature target. Minimum reporting should include time from collapse to ROSC, ROSC-to-cooling initiation, initiation-to-target, temperature measurement site, cooling method, time in target range, temperature variability, burden of shivering, sedative and neuromuscular-blocking exposure, duration of MTH, rewarming rate, and occurrence of rebound fever. Without these metrics, “33 °C” remains a protocol label rather than a reproducible physiological intervention [29].

The same applies to induction and rewarming performance. Hypothermia delivered slowly, maintained imprecisely, or followed by rebound fever is not equivalent to rapid, tightly controlled cooling with gradual, protocolized rewarming. These distinctions are often underrepresented in simplified discussions of TTM, yet they may be among the most important determinants of whether a biologically meaningful intervention has actually been administered.

Temperature strategy also affects neurological assessment. Hypothermia, shivering suppression, sedation, neuromuscular blockade, and delayed drug clearance may postpone awakening and confound early clinical examination. Therefore, any renewed evaluation of MTH should be inseparable from standardized, delayed, multimodal neuroprognostication. Prognostic decisions should not rely on a single early clinical sign, but on an integrated assessment using neurological examination, electrophysiology, biomarkers, and imaging after major confounders have been excluded.

## 7. What Should Current Evidence Mean for Practice?

Current evidence does not support the routine use of MTH in all comatose survivors of OHCA. However, it also does not establish that every form of hypothermia is ineffective across all clinical phenotypes and treatment settings. The most appropriate interpretation is therefore one of clinical equipoise regarding its potential role in carefully selected patients.

**Figure 1:**
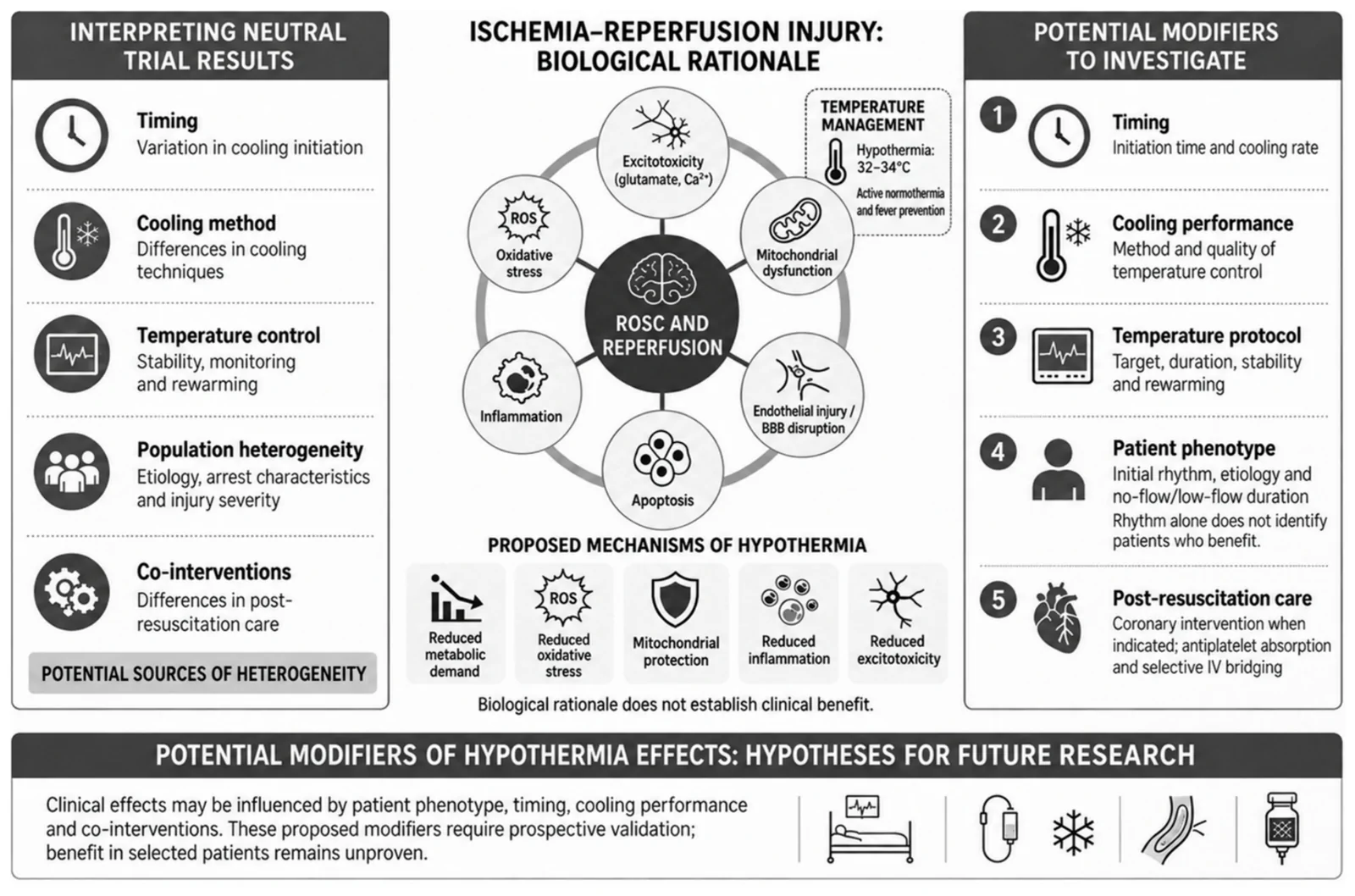
Potential modifiers of hypothermia effects: hypotheses for future research. Ischemia–reperfusion injury provides a biological rationale for temperature modulation after cardiac arrest and involves excitotoxicity, oxidative stress, mitochondrial dysfunction, inflammation, endothelial injury, and apoptotic signalling. The figure outlines potential modifiers of the clinical effects of mild therapeutic hypothermia, including the timing of initiation, cooling method and performance, temperature-control quality, patient phenotype, and post-resuscitation care, particularly coronary intervention when indicated and antiplatelet treatment. These factors may contribute to heterogeneity across trials, but they have not been established as modifiers of treatment benefit or as explanations for neutral trial results. Initial rhythm alone does not identify patients who benefit from hypothermia. This framework is intended to generate hypotheses for prospective evaluation; it does not establish efficacy in selected patient groups.

Active temperature control, continuous core-temperature monitoring, and strict fever prevention should remain the foundation of contemporary post-resuscitation care. If MTH is considered in an individual patient, it should be delivered within a high-quality, protocolized pathway that includes rapid assessment of the arrest phenotype, precise temperature control, careful haemodynamic support, structured neuroprognostication, and an adapted antiplatelet strategy in patients undergoing PCI (Figure 1).

Temperature management after cardiac arrest should therefore not be reduced to a binary choice between 33 °C and normothermia. It should be understood as a complex therapeutic strategy whose clinical effect may depend on patient phenotype, timing of initiation, quality of delivery, and interaction with other components of post-resuscitation care.

## 8. Limitations of This Viewpoint

This article is a clinical viewpoint and should not be interpreted as a systematic review. Accordingly, no formal systematic search or study-selection procedure was performed. The literature was selected to represent pivotal randomized trials, relevant meta-analyses, contemporary guidelines, and studies directly addressing the mechanistic and clinical arguments presented in this article. Much of the evidence supporting a phenotype-oriented approach to MTH is derived from mechanistic rationale, observational studies, post hoc analyses, and subgroup findings. Although these sources are valuable for generating hypotheses, they cannot establish clinical efficacy or support the routine use of MTH. The purpose of this viewpoint is therefore not to advocate the indiscriminate reintroduction of MTH, but to identify the clinical and procedural conditions under which it may warrant further evaluation in prospective trials.

## 9. Future Directions

The next step for this field is not to retest older questions in broader pragmatic populations, but to design trials that more accurately reflect the biology and technical demands of MTH. Future studies should focus on carefully selected OHCA phenotypes, particularly those in whom the biological rationale for neuroprotection is the strongest. Cooling should be initiated as early as possible after ROSC or even during resuscitation with clear separation of time-to-initiation and time-to-target metrics. A proposed framework for future confirmatory efficacy trials and complementary mechanistic studies, including patient selection, timing and delivery of cooling, comparator strategy, stratification, co-interventions, outcomes, and neurological prognostication, is summarized in Table 2.

The PRINCESS trial provided an important clinical test of whether cooling initiated during ongoing resuscitation could improve neurological outcomes. Transnasal evaporative intra-arrest cooling shortened the time to the target temperature but did not significantly increase the proportion of patients surviving with a favourable neurological outcome at 90 days in the overall study population [21]. These findings demonstrated the feasibility of initiating cooling before hospital arrival but did not establish the clinical efficacy of this strategy.

Building on this experience, the ongoing PRINCESS2 trial evaluates ultra-early therapeutic hypothermia initiated at the scene of OHCA using transnasal evaporative cooling, followed by standardized in-hospital temperature management. In contrast to PRINCESS, PRINCESS2 uses phenotype-oriented enrolment restricted to patients with an initial shockable rhythm. Its recently published pilot phase demonstrated high protocol adherence and an acceptable safety profile, supporting the feasibility of this approach [27,30]. The definitive results may help determine whether the neutral findings of previous trials reflect a true lack of clinical efficacy or whether the effect of cooling depends on patient phenotype, intervention timing, and treatment delivery.

High-performance cooling devices and methods should be used, temperature monitoring should be harmonized, and associated treatments, especially PCI and antiplatelet therapy, should be protocolized. In addition, future trials should place greater emphasis on the quality of cooling rather than merely on protocol assignment. An ideal next-generation trial should use an enrichment design rather than broad unselected enrolment. Eligibility should be based on predefined arrest phenotype and early injury severity, with stratification by initial rhythm, no-flow and low-flow intervals, lactate or pH, shock status, culprit coronary lesion, and need for mechanical circulatory support. The intervention should standardize cooling method, target temperature, duration, rewarming, shivering control, and antiplatelet strategy. Outcomes should include not only mortality and global functional scales, but also cognitive recovery, health-related quality of life, arrhythmias, bleeding, infection, vasopressor burden, and rebound fever.

## 10. Conclusions

The available evidence does not support a universal clinical benefit of MTH. Whether a clinically meaningful effect exists in selected patients and depends on phenotype, timing of initiation, or precision of delivery remains uncertain and requires prospective evaluation.

This is particularly important in acute coronary syndrome, where the potential neuroprotective effect of MTH intersects with invasive coronary treatment and with clinically important alterations in antiplatelet therapy.

Accordingly, the debate on MTH should remain open, but its next phase should be narrower, more clinically disciplined, and hypothesis-driven. Future studies should determine whether early, precisely delivered MTH can improve outcomes in patients with a defined neuroprotective window within an optimized post-resuscitation pathway.

## Figures and Tables

**Table 1:** Key clinical studies of hypothermia and temperature management after out-of-hospital cardiac arrest.

Study [Ref.]	Design and Population	Clinical Phenotype	Temperature Strategy	Cooling Timing and Method	Principal Outcome	Key Limitations/Safety	Relevance to the Current Viewpoint
Bernard et al., 2002 [5]	RCT; 77 comatose OHCA survivors	Predominantly VF; presumed cardiac cause	Hypothermia to 33 °C vs. conventional normothermic care	Cooling initiated early after ROSC; external cooling with ice packs	Favourable neurological outcome at discharge was more frequent with hypothermia	Small, highly selected population; control group without contemporary active fever prevention	Supports biological and clinical plausibility of early MTH in selected shockable-rhythm OHCA
HACA Study Group, 2002 [6]	Multicentre RCT; 275 comatose OHCA survivors	Witnessed VF arrest of presumed cardiac origin	32–34 °C vs. conventional care	Cooling initiated after hospital admission; external cooling	Favourable neurological outcome and survival with hypothermia	Selected VF population; conventional-care comparator differs substantially from contemporary temperature control	Established the original efficacy signal for MTH, but in a highly selected population and different treatment era
Kozinski et al., 2013 [16]	Observational; 32 comatose OHCA survivors; ACS-network implementation study	Comatose OHCA survivors integrated into an invasive ACS pathway	Protocolized MTH, generally 32–34 °C	MTH integrated with coronary angiography/PCI and structured post-arrest care	Network-based implementation was associated with improved clinical outcomes	Non-randomized design; potential confounding by improvements in PCI and overall post-arrest care	Suggests that the effect associated with MTH may depend on integration with the entire post-resuscitation pathway
Nielsen et al., TTM, 2013 [7]	International multicentre RCT; 950 unconscious OHCA survivors	Broad OHCA population of presumed cardiac cause; predominantly shockable rhythms	33 °C vs. active temperature control at 36 °C	Hospital-based; surface or intravascular cooling	No significant difference in mortality or neurological outcome	Heterogeneous cooling methods and timing; comparison of two actively controlled temperature strategies	Strong evidence against universal superiority of 33 °C; does not specifically address ultra-early, phenotype-selected MTH
Glover et al., 2016 [18]	Post hoc analysis of the TTM trial; 934 unconscious OHCA survivors	TTM population analysed according to cooling device	Temperature targets according to original TTM allocation	Intravascular vs. surface cooling	Intravascular cooling provided more precise temperature control; no definitive clinical outcome advantage demonstrated	Cooling method was not randomized; potential selection bias	Shows that identical nominal temperature targets may represent different delivered thermal exposures
Nürnberger et al., 2017 [19]	Secondary observational analysis of CIRC data; 263 OHCA survivors	OHCA patients receiving prehospital and/or in-hospital cooling	MTH vs. no cooling; timing-dependent comparison	Prehospital plus in-hospital vs. in-hospital-only cooling	Earlier/continuous cooling was associated with a survival signal	Non-randomized secondary analysis; substantial potential for residual confounding	Provides hypothesis-generating support for the importance of cooling initiation timing
De Fazio et al., 2019 [20]	Secondary analysis of the TTH48 trial; 352 OHCA survivors	Comatose OHCA patients undergoing TTM	Protocol-defined temperature control	Intravascular vs. surface cooling	Differences in temperature-control performance between cooling methods	Device allocation not randomized; no definitive evidence of clinical superiority	Supports standardization and reporting of cooling technology in future MTH trials
Nordberg et al., PRINCESS, 2019 [21]	International multicentre RCT; 677 OHCA survivors	Broad OHCA population undergoing advanced resuscitation; shockable and non-shockable rhythms	Transnasal evaporative intra-arrest cooling plus conventional systemic cooling vs. conventional systemic cooling alone	Selective brain cooling initiated during resuscitation in the prehospital setting	Shortened time to the target temperature but did not significantly improve favourable neurological outcome at 90 days in the overall population	Study stopped early for futility; heterogeneous arrest phenotypes; intervention differed from conventional whole-body MTH; no definitive overall efficacy signal	Demonstrated the feasibility of intra-arrest cooling but did not establish clinical efficacy; informed the more phenotype-oriented design of PRINCESS2
Lascarrou et al., HYPERION, 2019 [22]	Multicentre RCT; 584 OHCA survivors	Initial non-shockable rhythm; OHCA and in-hospital cardiac arrest	33 °C for 24 h vs. targeted normothermia at 37 °C	Cooling initiated after hospital admission using centre-specific methods	Favourable neurological outcome at 90 days was more frequent with hypothermia; mortality did not differ significantly	Small absolute number of favourable outcomes; fragile treatment effect; mixed OHCA and in-hospital population; potential influence of prognostication and withdrawal-of-care practices	Suggested a possible benefit in the non-shockable-rhythm phenotype, but required confirmation
Ratajczak et al., 2021 [23]	Observational cohort of 59 OHCA survivors treated with MTH	Mixed OHCA population	Protocolized MTH	Cooling after ROSC; induction time specifically evaluated	Outcome was primarily determined by age and neurological/injury severity; rapid cooling was not independently associated with better outcome	Small cohort; no normothermia comparator; confounding by severity of injury	Highlights the distinction between early initiation of cooling and rapid target achievement, the latter potentially reflecting severe neurological injury
Dankiewicz et al., TTM2, 2021 [8]	International multicentre RCT; 1861 comatose OHCA survivors	Broad OHCA population; predominantly shockable rhythms	33 °C vs. normothermia with active treatment of fever	Hospital-based; surface or intravascular temperature control	No reduction in 6-month mortality or poor functional outcome with 33 °C	Arrhythmias causing haemodynamic compromise were more frequent with hypothermia; heterogeneous population and intervention delivery	Strongest evidence against routine MTH in unselected OHCA; central evidence defining current equipoise
Simpson et al., 2022 [24]	TTM2 substudy; 1592 OHCA survivors	OHCA population included in TTM2	33 °C vs. normothermia	Cooling speed analysed in relation to treatment effect	Faster cooling did not significantly modify the effect of hypothermia	Secondary analysis; cooling speed may not fully reflect biologically relevant timing	Important counterargument to a simplistic “faster cooling is better” hypothesis
Sobczyk et al., 2024 [17]	Observational study; 54 OHCA survivors	Cardiac arrest occurring in the course of myocardial infarction	MTH vs. non-MTH management	Clinically determined; intravascular cooling relevant to reported findings	MTH was associated with better prognosis in selected patients	Non-randomized; residual confounding and subgroup effects	Supports further evaluation of an ACS-related phenotype rather than universal MTH
Taccone et al., 2024 [25]	Secondary analysis of TTM2; 600 OHCA survivors	HACA-like population with shockable rhythm	33 °C vs. normothermia (target ≤ 37.5 °C; cooling device initiated at ≥37.8 °C)	As in TTM2	No improvement in 6-month survival or functional outcome with hypothermia	Secondary analysis; arrhythmias more frequent with hypothermia	Strongly challenges the concept that shockable rhythm alone identifies patients likely to benefit from MTH
Levy et al., 2024 [26]	Exploratory analysis related to HYPO-ECMO; 159 OHCA survivors	Cardiac arrest/refractory cardiogenic shock requiring VA-ECMO	Hypothermia vs. normothermia strategy	High-intensity temperature management in the VA-ECMO setting	Exploratory findings suggesting possible phenotype-specific interaction	Highly selected critically ill population; not designed to establish routine post-OHCA MTH efficacy	Illustrates the need to study specific haemodynamic phenotypes separately from general OHCA populations
Dillenbeck et al., PRINCESS2 design, 2024 [27]	International multicentre randomized trial protocol; planned sample size: 996 OHCA survivors	OHCA with initial shockable rhythm	Ultra-early selective brain cooling plus standardized in-hospital temperature management vs. standard care	On-scene transnasal evaporative cooling	Definitive neurological outcome results pending	Ongoing trial; intervention differs from conventional whole-body MTH	Directly tests whether much earlier cooling in a phenotype-enriched population can alter clinical outcome
Taccone et al., HYPERION/TTM2 meta-analysis, 2024 [28]	Individual-patient-data meta-analysis of the HYPERION and TTM2 trials; 912 OHCA survivors	OHCA with initial non-shockable rhythm	Hypothermia at 33 °C vs. controlled normothermia	Temperature management according to the respective trial protocols	Hypothermia did not significantly reduce mortality or improve functional outcome	Post hoc pooled analysis; differences between trial populations and protocols; limited ability to identify small phenotype-specific effects	Does not confirm a benefit of 33 °C in OHCA with non-shockable rhythm and argues against rhythm alone as a sufficient selection criterion
Awad et al., 2025 [29]	Post hoc analysis of the TTM2 hypothermia arm; 930 OHCA survivors	OHCA patients assigned to hypothermia	Hypothermia according to TTM2 protocol	Intravascular vs. surface cooling	Intravascular cooling achieved faster and more precise temperature control; clinical superiority was not established	Non-randomized device selection; post hoc analysis	Supports the concept that cooling performance and delivered thermal exposure should be standardized in future trials
Dillenbeck et al., PRINCESS2 pilot, 2026 [30]	Randomized pilot phase of PRINCESS2; 100 OHCA survivors	Shockable-rhythm OHCA	Ultra-early selective brain cooling plus subsequent standardized temperature management	On-scene transnasal evaporative cooling	Demonstrated feasibility and high protocol adherence; efficacy assessment pending	Pilot phase not powered for definitive neurological efficacy	Demonstrates feasibility of ultra-early cooling and provides the platform for a definitive test of the timing hypothesis

**Abbreviations:** ACS, acute coronary syndrome; HACA, hypothermia after cardiac arrest; MTH, mild therapeutic hypothermia; OHCA, out-of-hospital cardiac arrest; PCI, percutaneous coronary intervention; RCT, randomized controlled trial; ROSC, return of spontaneous circulation; TTM, targeted temperature management; VA-ECMO, venoarterial extracorporeal membrane oxygenation; VF, ventricular fibrillation. **Note:** Temperature-management protocols differed substantially across studies with respect to patient selection, timing of initiation, cooling technology, target temperature, comparator strategy, duration of treatment, and rewarming. Consequently, assignment to “hypothermia” should not be interpreted as representing a uniform physiological intervention.

**Table 2:** Proposed research framework for future studies of mild therapeutic hypothermia after OHCA.

Domain	Confirmatory Efficacy RCT	Mechanistic/Thermal-dose Study	Key Question
Population	Enriched OHCA population with presumed cardiac aetiology and a potentially modifiable neurological injury phenotype; initial rhythm, witnessed status, no-flow/low-flow duration, pH/lactate, and shock status should inform eligibility	Broader phenotype range allowing assessment of interactions between baseline injury severity and physiological response to cooling	Which clinical phenotype retains a potentially modifiable neuroprotective window?
Timing of cooling	Cooling initiated as early as feasible after ROSC; prehospital versus later initiation should be directly evaluated where appropriate	Recording of key cooling intervals, serial prehospital core temperatures, and factors affecting passive cooling	How do intentional and unintended cooling affect thermal exposure and biological response?
Temperature protocol	Prespecified MTH target and duration, controlled rewarming, and protocolized fever prevention, compared with contemporary active normothermia	Continuous characterization of temperature trajectory, time in target range, temperature variability, rewarming, and rebound fever	What temperature exposure constitutes an effective and reproducible thermal dose?
Cooling and temperature monitoring	Standardized high-performance cooling technique and prespecified core-temperature measurement site across participating centres	Comparison of cooling technologies and measurement sites during induction, maintenance, and rewarming	How do cooling technology and temperature monitoring influence the delivered thermal dose?
Comparator	Active normothermia with continuous core-temperature monitoring and protocolized fever prevention	Identical background care with detailed physiological monitoring	Does precisely delivered MTH improve outcomes compared with contemporary active normothermia?
Stratification	Prespecified stratification by initial rhythm, arrest duration/injury severity, cardiogenic shock, eCPR, culprit coronary lesion, and mechanical circulatory support	Interaction analyses across predefined physiological and treatment phenotypes	Which clinical and treatment-related factors modify the biological or clinical effect of MTH?
Co-interventions	Standardized post-resuscitation care, including haemodynamic management, sedation/shivering control, coronary strategy, and antithrombotic treatment when PCI is performed	Assessment of interactions between cooling, haemodynamics, drug exposure, platelet inhibition, and organ function	To what extent is the effect of MTH modified by accompanying therapies?
Efficacy and safety outcomes	Primary: survival with favourable neurological/functional outcome at a prespecified long-term time point, preferably 6 months. Secondary: mortality, cognitive recovery, health-related quality of life, and organ-support requirements. Safety: arrhythmias, bleeding, infection, haemodynamic deterioration, vasopressor requirements, metabolic disturbances, and delayed awakening	Biomarkers of neurological injury and systemic response correlated with thermal exposure and adverse physiological effects	Does MTH provide clinically meaningful neuroprotection, and does any benefit outweigh its physiological cost?
Neurological prognostication	Standardized, delayed, multimodal prognostication after relevant confounders from hypothermia, sedation, and neuromuscular blockade have been minimized	Assessment of the influence of thermal exposure on awakening and neurological prognostic markers	Can MTH-related effects on awakening or prognostic markers bias outcome assessment and treatment-withdrawal decisions?

**Abbreviations:** eCPR, extracorporeal cardiopulmonary resuscitation; MTH, mild therapeutic hypothermia; OHCA, out-of-hospital cardiac arrest; PCI, percutaneous coronary intervention; RCT, randomized controlled trial; ROSC, return of spontaneous circulation. **Note:** Specific thresholds for no-flow and low-flow duration, pH, lactate concentration, cooling rate, and other enrichment variables are intentionally not proposed because current evidence does not allow the reliable identification of treatment-effect cut-offs. These parameters should be prospectively prespecified, and whenever possible, evaluated as continuous variables before being incorporated into future enrichment strategies.

## Data Availability

No new data were created or analyzed in this study. Data sharing is not applicable to this article.
